# The Risky Side of Creativity: Domain Specific Risk Taking in Creative Individuals

**DOI:** 10.3389/fpsyg.2017.00145

**Published:** 2017-02-03

**Authors:** Vaibhav Tyagi, Yaniv Hanoch, Stephen D. Hall, Mark Runco, Susan L. Denham

**Affiliations:** ^1^Cognition Institute and School of Psychology, Plymouth UniversityPlymouth, UK; ^2^Department of Educational Psychology, University of Georgia, AthensGA, USA

**Keywords:** creativity, risk taking, domain specificity, social risks, DOSPERT

## Abstract

Risk taking is often associated with creativity, yet little evidence exists to support this association. The present article aimed to systematically explore this association. In two studies, we investigated the relationship between five different domains of risk taking (financial, health and safety, recreational, ethical and social) and five different measures of creativity. Results from the first (laboratory-based) offline study suggested that creativity is associated with high risk taking tendencies in the social domain but not the other domains. Indeed, in the second study conducted online with a larger and diverse sample, the likelihood of social risk taking was the strongest predictor of creative personality and ideation scores. These findings illustrate the necessity to treat creativity and risk taking as multi-dimensional traits and the need to have a more nuanced framework of creativity and other related cognitive functions.

## Introduction

The great sculptor, painter, and architect, Michelangelo frequently depicted the sensual form of human bodies in religious contexts such as in his masterpiece, ‘David’. In one incident, his fresco, ‘The last judgment’ was highly criticized by the then Pope’s master of ceremonies since it “depicted…nude figures, exposing themselves" (Land, 2013). Michelangelo responded by painting the official’s face into the mural and covering his nude figure with a snake. Anecdotes such as this have provided support for the notion that creative individuals are risk takers. In his seminal work, McClelland proposed that ‘a calculated risk’ is an important aspect of scientific performance (McClelland, 1963). Other writers have expressed similar views (Sternberg and Lubart, 1995; Sternberg, 1997; Runco, 2015; Steele et al., 2016), calling a creative act a risk (Haefele, 1962), as well as referring to the willingness of creative individuals to risk the uncertainty of the unknown (Getzels and Jackson, 1962). Despite these suggestions, most of the literature is speculative in nature and little empirical data exists to support such claims. To bridge this gap, we investigated whether creativity is associated with risk taking; more importantly, we specifically examined the link between creativity and risk taking in five different domains or content areas.

Early scientific investigation of risk taking in the context of creativity was an exploratory study conducted by Merrifield et al. (1961). They found a significant correlation between participants’ associational fluency (a measure of divergent thinking based assessment of creativity) and their score on adventure as a component of risk taking. Building on this work and McClelland’s speculations, Pankove and Kogan (1968) conducted a study among fifth grade children. They measured creative ability with divergent thinking tasks which involve generating as many solutions to open-ended problems as possible. Risk taking was measured by a variety of behavioral tasks which were hypothesized to involve certain degrees of risk. The researchers reported a significant relationship between risk taking and creativity only when risk was measured by one of the tasks. The other measures of risk taking did not yield any evidence of a relationship between risk taking and creative ability.

Other researchers have investigated the relationship between creativity and risk taking using related but indirect measures. Fleming and Weintraub (1962), for instance, found an inverse relationship between rigidity and intolerance of ambiguity as a measure of risk and creativity, in elementary school children. Similarly, Kurtzman (1967) measured adventurousness as a personality characteristic and used several tests from the *kit of reference tests for cognitive factors* to measure creativity (Ekstrom et al., 1976). He did this study on ninth grade girls and found that highly creative girls were significantly more adventurous than the less creative. Nicolay (1966), on the other hand, reported a lack of significant correlation between risk taking and creativity in female undergraduates.

A growing body of recent literature has also reported mixed findings when measuring risk taking and creativity under specific conditions. For example, Ivcevic and Mayer (2006) measured three dimensions of creative behavior – creative life style, performing arts and intellectual achievement using the *life report questionnaire* (Ivcevic and Mayer, 2009). Monetary risk taking was also measured using the *Risk taking Personality Inventory* which measures risk taking in five domains. They found that the individuals who scored high on intellectual achievement exhibited high risk taking tendencies in the professional and financial areas. In contrast, Erbas and Bas (2015) did not find any significant correlations between the creative ability as measured by the *Creative Ability in Mathematics Test* and academic risk taking as measured by the *Academic Risk Taking Scale* among ninth graders. The aforementioned studies clearly demonstrate a lack of consensus regarding the relationship between risk taking and creativity. Some report a small positive correlation while others find no significant relationship between various measures of creativity and risk taking. As pointed out by Strum (1971), this lack of consensus may be attributed to the specific methods used to measure creativity and risk taking, diversity in the definition of risk taking, differences in the number of participants and other aspects of demographics, including cultural differences. Such varied and differentially motivated research warrants an obvious, yet important question: Are creative individuals high risk takers?

In order to answer this question and to address earlier shortcomings, in the current investigation we used a wider range of standardized performance and questionnaire based instruments to obtain comprehensive measures of creativity and risk taking. Measuring creativity has been an exceptionally challenging task throughout the history of creativity research. Although numerous attempts have been made to measure different dimensions of creativity, they are marred with criticism. Past studies which aimed to explore the relationship between creativity and risk taking have equated creativity to measures such as associational fluency, divergent thinking, tolerance of ambiguity, creative lifestyle or intellectual achievements. Each of these measures only provide a narrow insight into some aspects of creativity. Contrary to the previous studies, we treated creativity as a multidimensional trait and used both biographical and behavioral measures of creativity [creative personality, creative achievements in multiple domains, creative ideation, problem solving, and divergent thinking] in large participant populations, including both student and non-student samples under different test conditions. This holistic approach is in line with recent studies advocating the use of a large, diverse group of measures to capture creativity (Eisenman, 1969; Cropley, 2000; Fields and Bisschoff, 2013). We propose that given the multidimensional nature of creativity, a holistic measurement will be more effective in capturing this construct.

In conjunction with the creativity measures, a gambling task called Roulette Betting Task (RBT) was employed to measure risk taking (Studer and Clark, 2011). This task has been shown to be a simple yet effective tool for measuring variables related to risk taking. However, while the gambling tasks provide an effective method of identifying risk taking in the financial domain, they do not guarantee that the resultant measures are applicable more generally. Indeed, to better capture the complex nature of risk taking, several researchers have argued for the need to measure risk taking in several domains. Slovic (1964), one of the early advocates of this idea, questioned the assumption that financial risk taking is a robust predictor of other types of risk taking. Following Slovic’s idea, other researchers have developed measures intended to examine risk taking tendencies in more than one domain. Weber et al. (2002), identified five domains of risk taking and developed a questionnaire called *DOSPERT* (*Domain Specific Risk Taking Questionnaire*) based on their results (Blais and Weber, 2006). There is now sufficient evidence, from studies with diverse populations (Hanoch et al., 2006; Rolison et al., 2014), to support Slovic’s argument, demonstrating the need to investigate risk taking across multiple domains.

Domain specificity is particularly relevant for our understanding of risk in relation to research in creativity because risk taking in some domains appears to be more pertinent to creativity than others. For example, it is possible that some domains of risk taking (such as social or recreational) are more closely associated with creativity than others (for instance, gambling). Sternberg (1997) provided support to this notion by referring to the importance of ‘sensible’ risk taking in creativity. He emphasized that the risk of being ‘different’ is more important in creativity than risks that endanger limbs or life. In line with these views, the current study aimed to systematically investigate the association between domain specific risk taking and a holistic measurement of creativity. We predicted that specific domains of risk taking such as social risk taking (i.e., the willingness to challenge norms) would show a positive association with creativity. We investigated these associations in a laboratory-based study which included behavioral and questionnaire based measures of creativity and risk taking. This was followed by an online study with a larger and more diverse group of individuals in order to explore the wider validity of the findings. To our knowledge, this is the first study to systematically investigate the relationship between domain specific risk taking and creativity.

## Study 1

All the data for the first study were collected under laboratory based conditions from participants based in the UK.

### Method

#### Participants

Sixty-four volunteers (34 female, *M*_age_ = 23 years, *SD* = 4.36), were recruited from a paid participant pool and via posters across the university. All participants were paid 8 for their participation. The study received ethical approval from the research ethics committee at the researchers’ university.

#### Measures of Risk Taking

##### (i) Roulette Betting Task

The RBT was used as a behavioral measure of risk taking alongside the questionnaire based measure (DOSPERT). In this task, participants were presented with a wheel containing 10 segments or ‘pockets’ on a computer screen. Each pocket was either red or blue colored. Through verbal and written instructions, participants were informed that the blue pockets were associated with wins while red with losses. In each trial, they were presented with three boxes indicating the available bet options – low, medium, and high. Participants were instructed to select one bet, and upon selection, the wheel spun for a variable amount of time (3–3.5 s) before randomly stopping on one of the 10 pockets. Finally, a text feedback indicated whether they won or lost the money. The ratio of blue to red colored pockets determined the probability of winning. This probability was varied at three levels – small (40% chance of winning), medium (60%), and large (80%). The probabilities of rewards and magnitude of the bet options were randomized across all trials. In total, 100 trials were presented to each participant. Before the commencement of the task, they were informed that the highest score obtained by one of the participants on this task would be converted into a monetary donation to a local charity of their choice.

##### (ii) Domain Specific Risk Taking Scale (DOSPERT)

DOSPERT is a standardized questionnaire which contains 30 questions related to five different domains of risky behaviors (Ethical, Financial, Health/Safety, Recreational, and Social) (Blais and Weber, 2006). Each domain contains six questions and individuals rate the likelihood of engaging in risky activities on a seven point Likert scale. Higher values on the scale represent higher chances of engaging in risk taking. Sample items on the scale include “Disagreeing with an authority figure on a major issue” (social), “Passing off somebody else’s work as your own” (ethical), “Driving a car without wearing a seat belt” (health/safety), “Bungee jumping off a tall bridge” (recreational), and “Betting a day’s income at a high-stake poker game” (financial). The scale ranges from ‘extremely likely’ to ‘not likely at all.’

DOSPERT also measures perception of risk (from ‘extremely risky’ to ‘not at all’) and expected benefits (from ‘great benefits’ to ‘no benefits at all’) on seven point scales. ‘Risk perception’ responses measures individuals’ gut-level assessment of risk. On the other hand, ‘expected benefits’ responses evaluate the degree of benefit that an individual sees in each risky activity.

#### Measures of Creativity

##### (i) Divergent Thinking Task

The alternate uses task (AUT) measures divergent thinking in individuals (Guilford, 1967). This task contains the names of several common household items (such as eyeglasses or a shoe) and participants were presented with these, one at a time. They were instructed to generate as many unusual uses as possible for each item. There was no limit on the time individuals took to record their responses; however, they were instructed to spend at least 2 min on each object. This time limit was carefully monitored by the experimenter. We administered the test using a computerized version of the test items and hence instructed our participants to type in their responses.

##### (ii) Compound Remote Associates Task (c-RAT)

The compound remote associates task is based on the original task (Mednick, 1968) and provides a wide variety of remote associates problems (Bowden and Jung-Beeman, 2003). In each of these problems, participants are presented with three words which are associated with a target word. The task for participants is to find the target word. Every correct response increases the total score by one. We selected thirty items from a set of 144 items provided in c-RAT. All of the items were randomly selected from a uniform distribution across all the difficulty levels. The task was presented on a computer and participants had 15 s to type in their responses in each trial.

##### (iii) Creative Achievement Questionnaire (CAQ) and Runco’s Ideational Behavioral Scale (RIBSs)

Creative Achievement Questionnaire assesses achievement across 10 different domains of creativity: music, visual arts, architecture, scientific discovery, culinary arts, dance, theater and films, inventions, writing and humor (Carson et al., 2005). It is a self-report measure in which participants are asked to report their achievements in these 10 domains.

Runco’s Ideational Behavioral Scale is a self-report measure of creativity which measures creative ideation (Runco et al., 2001). Participants are asked to report how frequently they generate ideas on a five-point scale (from never to daily) in response to nineteen different questions relating to their day-to-day ideas and ideation ability.

##### (iv) Creative Personality Scale (CPS)

Developed by Gough (1979), the creative personality scale (CPS) presents participants with a set of thirty adjectives. Gough identified a list of adjectives which comprise a creative personality and contrasted them with adjectives which do not. Participants indicate the adjectives that apply to them via a checklist. These adjectives are then scored positively or negatively according to a standardized scoring key to calculate the composite creative personality score.

#### Self-Reports

In addition to the tasks and questionnaires, participants were asked to rate themselves on ‘how creative they are’ and ‘how risk taking they are’ on five-point Likert scales.

#### Procedure

All the tasks were presented using Psychopy2 (Peirce, 2007; Peirce, 2009). Questions and ratings for all the questionnaires were presented in an online survey web service with no restriction on time (SurveyMonkey Inc.)^1^. All the tasks and questionnaires were spread across two, 1-h sessions for each participant. Each session consisted of tasks followed by questionnaires and the order of tasks and questionnaires was randomized across participants.

#### Data Analysis

The RBT provided two measures of financial risk taking behavior. The average bet amount across all the decision trials provided a measure of financial-gambling related risk taking for each participant (average bet). The change in bet amounts as a function of the probability of winning (the slope of the best line of fit), provided a measure of adjustment to risk (gambling risk adjustment). For creative thinking tasks, standard measures of analysis were used. The divergent thinking task allowed a measurement of originality and fluency; originality was the average statistical infrequency of the ideas and fluency score was the total number of ideas generated by each participant. Scores on the compound remote associates task were obtained by a summation of all the correct responses.

All the questionnaire scores were calculated using standard scoring keys and scoring procedures provided with respective questionnaires. We followed the suggestions provided in Silvia et al. (2012) and calculated nominal scores for each domain in the CAQ in order to avoid conducting further statistical analysis on skewed raw scores. The threshold for calculating the nominal scores were 0 (=0), 1 to10 (=1), and more than 10 (=2). A total CAQ score across all the domains was used in subsequent analyses. CAQ provided a creative achievement score while RIBSs provided a score of ideation fluency. Finally, DOSPERT provided scores for risk taking in each of the five risk domains.

Performance on all the tasks and scores from questionnaires were entered in a multiple correlational analysis where each factor was pairwise correlated with all the other factors. We chose to perform Bayesian correlation analysis on our data since it allowed us to analyze the probability of both null (Bayes Factor BF_01_) and alternate hypothesis (Bayes Factor BF_10_) testing. We used a stringent threshold of Bayes factors higher than 30 for determining the *very strong evidence* in favor of the presence of correlations. In order to interpret our results, we followed Jeffreys’ suggestions (Jeffreys, 1961; Jarosz and Wiley, 2014), which provide an easy to interpret table of Bayes factors. In short, Bayes factor (BF_XY_) from 10 to 30 suggests a strong evidence for X; BF_XY_ from 30 to 100 suggests a very strong evidence for X and BF_XY_ greater than 100 is decisive for X. Moreover, a non-informative, uniform prior with a beta prior width of 1 was used throughout the analysis. An open source statistical analysis software called JASP (JASP Team, 2016) was used to conduct all the statistical analyses.

### Results

Fluency and originality scores on the divergent thinking task did not yield strong evidence of correlation with either task based or DOSPERT based measures of risk taking (BF_10_ < 3.5 for average bets, adjustment of bets, social, ethical, financial, health-safety and recreational risk likelihood, perception and benefits). There were also no statistically supported correlations between scores of divergent thinking and other measures of creativity (BF_10_ < 1.6 for CPS, RIBS and CAQ). Similarly, c-RAT scores did not show any supported correlations with either measures of risk taking (BF_10_ < 0.4) or with other measures of creativity (BF_10_ < 0.43).

**FIGURE 1:**
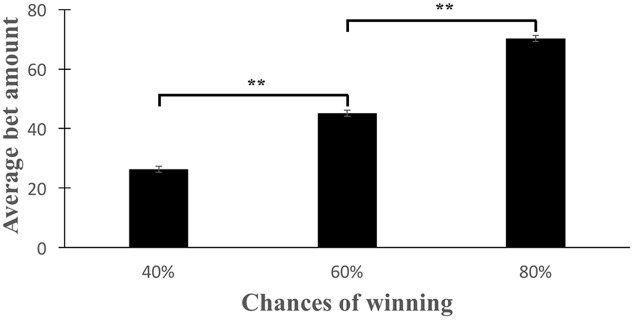
**Adjustment to risk.** Average bets selected showed a linear increase as the probability of winning increased (^∗∗^*p* < 0.001 in a pairwise *t*-test).

A paired samples *t*-test was conducted to compare the average bet placed in each probability condition. We found a significant linear increase in the average bet as the probability of winning increased (*p* < 0.001 for the within subject linear effect; non-significant for the within subject quadratic effect) (**Figure 1**). There was a significant difference in the average bets placed in 40% (*M*_bets_ = 26.26, *SD* = 8.36) and 60% (*M*_bets_ = 45.17, *SD* = 12.60) probability trials [*t*(63) = -12.27, *p* < 0.001] as well as between 60 and 80% (*M*_bets_ = 70.21, *SD* = 11.94) probability trials [*t*(63) = -14.58, *p* < 0.001].

Neither measure of financial risk taking behavior as measured by the gambling task showed strong evidence of correlations with any measure of creativity (BF_10_ < 0.6 for CPS, RIBS, CAQ, Fluency and Originality scores). Similarly, there was a lack of supported correlation between the likelihood of risk taking in the financial-gambling domain as measured by DOSPERT and the available measures of creativity (BF_10_ < 2.6). Following the same trend, we found that the likelihood of risk taking in recreational, financial-investment, health and safety and ethical domains also showed no supported correlations with measures of creativity (BF_10_ < 2.1). In contrast, scores on CPS (Pearson’s *r* = 0.42, BF_10_ = 49.4), CAQ (Pearson’s *r* = 0.46, BF_10_ = 233.14) and RIBS (Pearson’s *r* = 0.4, BF_10_ = 32.19) demonstrated strong evidence of correlations with the likelihood of risk taking in the social domain (**Table 1**).

**Table 1:** Domain specific risk taking and creativity: Study 1.

	Gambling		Likelihood of taking risks					
Creativity	Average bets	Risk adjustment	Social	Recreational	Financial (Gambling)	Financial (Investment)	Health and Safety	Ethical
**CPS**	***-0.01***	***-0.007***	***0.42^∗^***	***0.22***	***-0.04***	***0.03***	***0.18***	***0.18***
	0.16	0.16	49.4	0.675	0.163	0.16	0.422	0.403
**CAQ**	***-0.09***	***0.02***	***0.47^∗∗^***	***0.24***	***0.10***	***0.18***	***0.20***	***0.18***
	0.2	0.16	233.149	0.943	0.215	0.408	0.524	0.422
**RIBS**	***-0.20***	***-0.11***	***0.40^∗^***	***0.18***	***0.27***	***0.29***	***0.29***	***0.28***
	0.55	0.22	32.195	0.436	1.511	2.073	2.143	1.947

*Correlation table with Pearson’s correlation coefficients (in bold, italics) and their respective Bayes factors underneath them. Statistically supported correlations are marked (^∗^BF_10_ > 30, ^∗∗^BF_10_ > 100). CPS, Creative Personality Scale; CAQ, Creative Achievement Questionnaire; RIBS, Runco Ideational Behavioral Scale.*

Perception of risks and expected benefits did not show supported correlations with CPS, CAQ, or RIBS in any domain of risk taking (BF_10_ < 1.7). Only exception to this trend was a correlation between CAQ scores and expected benefits in the social domain (Pearson’s *r* = 0.45, BF_10_ = 162.7).

### Discussion

The results from this study demonstrate a strong link between risk taking in the social domain and personality and biographical inventory based measures of creativity. Other domains of risk taking were not significantly associated with any measure of creativity. Social risk taking is particularly interesting to investigate in the context of creativity. Creative individuals often present their ideas and creative products to social groups, for evaluation, appreciation, or criticism. This activity involves a high level of social risk especially since it entails the possibility of the creative idea or product being rejected by some, or all the individuals forming the social group.

Most participants reported that cRAT was extremely difficult and that they could not solve most cRAT problems in the time limit of 15 s. This was reflected in their scores, the maximum number of problems solved was 15 (out of 30). Accordingly, cRAT scores were removed from the subsequent analysis. Surprisingly, despite the widespread use of divergent thinking tasks as a proxy measure of creativity, divergent thinking scores showed no supported correlation with measures of risk taking and they were also not correlated with other measures of creativity. These results add to a plethora of literature questioning the appropriateness of the established divergent thinking based measures of creativity.

## Study 2

Given the relatively smaller sample size and homogeneous group of participants in Study 1, it is possible that participants’ creative achievements, ideation and personality were restricted by their experiences. Consequently, we ran a second study on a large and more diverse group of participants living in the USA.

### Method

#### Participants

Four hundred and seventeen participants (*M*_age_ = 36 years, *SD* = 12.26, 223 female) took part in this study for monetary compensation on a popular survey platform called Mechanical Turk (Buhrmester et al., 2011).

This study incorporated CAQ, RIBSs, CPS, DOSPERT, and self-reports (refer to Study 1 for details). It was self-paced and on average lasted for less than 30 min. In a manner similar to Study 1, we performed a multiple pairwise correlation analysis. Additionally, we were interested in investigating the degree to which risk taking in each of the five domains would predict measures of creativity. All scores from the questionnaires and self-reports were included in the correlation analysis which consequently informed the regression model. Finally, we performed additional analysis in order to find the effect of gender on creativity.

### Results

There were no differences between male and female groups on any scale of creativity. A multiple pairwise Bayesian correlation analysis showed that self-reports of risk taking showed strong evidence of correlations with the likelihood of risk taking in all the domains (BF_10_> 30 for all domains, Pearson’s *r* for social = 0.22, recreational = 0.54, financial/gambling = 0.35, financial/investment = 0.41, health/safety = 0.42, ethical = 0.37). Additionally, self-reports of risk taking were correlated with self-reports of creativity (Pearson’s *r* = 0.31, BF_10_ > 100), CPS (Pearson’s *r* = 0.29, BF_10_ > 100) and RIBS (Pearson’s *r* = 0.31, BF_10_ > 100) (**Table 2**).

**Table 2:** Domain specific risk taking and creativity: Study 2.

Creativity	Risk	Likelihood of taking risks					
Measures	Self-reports	Social	Recreational	Financial (gambling)	Financial (Investment)	Health and Safety	Ethical
**CPS**	***0.29^∗∗^***	***0.33^∗∗^***	***0.26^∗∗^***	***0.03***	***0.24^∗∗^***	***0.13***	***-0.003***
	4.917e+6	1.500e+9	118945.7	0.074	13533.62	2.129	0.061
**CAQ**	***0.16***	***0.15***	***0.20^∗∗^***	***0.13***	***0.16***	***0.16***	***0.14***
	9.484	6.141	228.7	2.095	15.35	14.95	3.385
**RIBS**	***0.31^∗∗^***	***0.29^∗∗^***	***0.26^∗∗^***	***0.22^∗∗^***	***0.25^∗∗^***	***0.21^∗∗^***	***0.14***
	6.857e+7	6.972e+6	88645.4	1753.16	55209.14	677.956	2.922

*Correlation matrix with Pearson’s correlation coefficients (in bold, italics) and their respective Bayes factors underneath them. Statistically supported correlations are marked (^∗∗^BF_10_ > 100). CPS, Creative Personality Scale; CAQ, Creative Achievement Questionnaire; RIBS, Runco Ideational Behavioral Scale.*

Measures of creativity and that of risk taking showed satisfactory internal consistency (Cronbach’s alpha for RIBS = 0.89, CPS = 0.77, CAQ = 0.62, social likelihood = 0.76, recreational likelihood = 0.84, financial/gambling likelihood = 0.91, financial/investment likelihood = 0.82, health/safety likelihood = 0.75 and ethical likelihood = 0.78). Pairwise correlations for a linear relationship of the likelihood of social risk taking with CPS and RIBS demonstrated strong statistical evidence (**Table 2**). The likelihood of taking recreational risks was found to be show supported correlations with all three measures of creativity while financial (investment) related risk was correlated with CPS and RIBS. None of the measures of creativity showed a supported correlation with risk perception in any domain (BF_10_ < 4.2). Similarly, CPS did not show any supported correlation with expected benefits in any domain (BF_10_< 0.6). Finally, RIBS showed supported correlations with expected benefits only in the social (Pearson’s *r* = 0.26, BF_10_> 100) and recreational domain (Pearson’s *r* = 0.25, BF_10_ > 100).

We ran three linear regression models each predicting creative personality (CPS), ideation (RIBS), and achievements (CAQ) using the likelihood of risk taking in each of the domains as predictors. The method used to build these models involved entering all the domains of risk at the same time. Additional stepwise methods of entering the domains of risk yielded the same result. Only the likelihood of risk taking in the social domain significantly predicted both creative personality and ideational ability [CPS: *F*(6,410) = 12.83, *p* < 0.001, *R*^2^ = 0.16, standardized coefficient for social risk taking = 0.237, *p* < 0.001; RIBS: *F*(6,410) = 12.05, *p* < 0.001, *R*^2^ = 0.15, standardized coefficient for social risk taking = 0.243, *p* < 0.001]. None of the other domains of risk taking were significant predictors of these creativity measures. None of the domains of risk taking predicted CAQ scores significantly [*F*(6,410) = 4.04, *p* < 0.001, *R*^2^ = 0.06, social *p* = 0.076, recreational *p* = 0.12, financial 0.39, health-safety *p* = 0.97 and ethical *p* = 0.56].

### Discussion

Results from this study corroborated the results from Study 1, thus confirming a clear association between social risk taking and personality and biographical inventory based measures of creativity. Interestingly, additional correlations were observed with other domains of risk taking in this study such as recreational, financial, and health-safety. Notably, the coefficient values for correlations between social risk taking and CPS as well as with RIBS decreased and stabilized in this study due to an increase in the sample size. This effect has been investigated in greater detail in previous studies. For instance, Schönbrodt and Perugini (2013) showed that for smaller sample sizes (such as in Study 1), Pearson’s coefficients fluctuate considerably and sometimes even change signs. However, with increasing sample size, the correlation coefficients decrease until they finally stabilize at a sample size of 200–250. Therefore, the larger sample size in Study 2 provided confidence required for the statistically supported results. Moreover, a multiple linear regression analysis showed that only social risk taking is a significant predictor of the ideation and personality based measures of creativity. Other domains of risk taking did not predict any measure of creativity in this study.

## General Discussion

“I am always doing that which I cannot do, in order that I may learn how to do it.” This quote by the creative polymath Pablo Picasso is one of the many, that identify the importance of taking risks in creativity. Previous scientific literature investigating the association between creativity and risk taking has reported mixed findings, mainly due to the differences in the size and type of participant sample and the specific instruments employed to measure risk taking and creativity (Strum, 1971). Most of these studies have reported measuring related but indirect variables; for instance, adventurousness for risk taking and divergent thinking for creativity. Previous reports have also been limited by their differentially motivated approaches; many sought out to investigate factors such as personality traits (Ivcevic and Mayer, 2006), promotion and prevention cues (Friedman and Förster, 2001), academic risk taking (Strum, 1971) or mathematical creativity (Erbas and Bas, 2015). In contrast, the current investigation was aimed at investigating the relationship between risk taking and creativity using a variety of behavioral, biographical, and personality based measures. The motivation for the current study specifically led us to ask the following question – Is risk taking generally associated with creativity or is this association domain specific?

The results from the first study indicated that among the six domains of risk taking, only social risk taking shows strong evidence for correlations with CPS, ideation, and creative achievements. None of the other domains of risk taking, as measured by the gambling task and risk taking questionnaire showed a statistically supported correlation with any of the measures of creativity. These results corroborate Sternberg’s idea of ‘sensible’ risk taking in creativity. He proposed that some domains of risk taking are more pertinent to creativity (for instance, the idea of being socially ‘different’) than others such as health and safety (risk of losing limbs or life) (Sternberg, 1997). Presenting a radical idea to a social group, unveiling a new artwork at an exhibition, publishing a collection of stories or poems and many other forms of social interactions involve a high degree of risk. All of the aforementioned acts are risky since there is always some uncertainty associated with the social evaluations. These creative acts thus require individuals who are willing to take risks in the social domain.

The second study was based on a much larger sample size and a diverse group of participants. The larger sample size also provided the statistical confidence required for the regression models. The results from this study demonstrated that social risk taking was the only statistically significant predictor of the measures of creativity. This provided support for our initial findings that creative individuals are more likely to take risks exclusively in the social domain.

The lack of correlation between financial risk taking in the gambling domain and measures of creativity is particularly important to discuss, since in most studies of risk taking, performance on gambling tasks is often equated to a general tendency toward risk taking. Our results from both the performance on the gambling task and scores from the questionnaires point toward the same direction; risk taking in the financial-gambling domain is not related to creativity. These results provide further evidence for the argument that the association between risk taking and creativity is domain specific.

Relationships between risk taking in the other domains (such as ethical) and creativity have been studied in specific scenarios such as deception (Gino and Ariely, 2012; Mai et al., 2015). For instance, Gino and Ariely (2012) reported that individuals with creative personalities cheated more than others in a deception task. Additionally, priming individuals to think creatively led them to be more likely to exhibit unethical behavior. We did not find support for these findings in our study. Both studies in the current research indicated that the likelihood of taking ethical risks is not related to measures of creativity. Niepel et al. (2015) recently criticized the study by Gino and Ariely, suggesting that due to the artificial nature of the deception tasks, participants were not only presented with the opportunity to behave dishonestly but they were also tempted to do so. They reported that self and teachers’ reports of creativity in a sample of students are positively linked to ethical decision making (as opposed to the negative associations found previously). Additionally, they reported that in the long term, creativity was not a general predictor of ethical decision making. Given the current scientific evidence, it is difficult to draw a strong conclusion based on these mixed findings and the question of the relationship between ethical risk taking and creativity remains unanswered.

We found mixed results with creative achievement scores in our study. While CAQ scores were significantly correlated with social risk taking in Study 1, we did not find this in our larger, diverse group of participants in Study 2. Additionally, none of the domains of risk taking were significant predictors of CAQ scores in the regression model. Inconsistency in the results may arise from the scoring structure of CAQ. Scores from this questionnaire are known to be highly skewed and several researchers have suggested using a nominal scoring procedure to avoid using raw scores (Silvia et al., 2012). Although, we have adopted this approach in our data analyses to limit the skewness in the scores, there are limitations to these correctional procedures and these are amplified as the datasets get larger. Consequently, it might have resulted in the differences in the two datasets. Future research could shed light on this association by using different measures of creative achievements (e.g., An and Runco, 2016; Paek et al., 2016).

Interestingly, unlike the questionnaire-based measures, the task-based measures of creativity did not correlate with risk taking (nor did they correlate with other measures of creativity). The tasks of creativity, such as the AUT measure divergent thinking, a component of creativity. Divergent thinking has been theorized as an important dimension of creativity, however, it doesn’t comprise all of it (Baer, 2011). Moreover, divergent thinking tasks aim to measure creativity in a very short time period. From our results, it seems likely that attitudes of risk taking in the social domain are related to biographical and personality based measures of creativity (creative personality, ideation, or achievements) as opposed to the task based measures.

### Limitations and Future Directions

Although the present research has shown that there is a significant association between creative personality and social risk taking, this study did not aim to explore the causal link between them. Previously, Dellas and Gaier (1970) have suggested that it is the personality traits which affect creative behavior, rather than the reverse. Future studies could explore the possibility of manipulation of social risk taking and investigating its effects on creativity. Additionally, external factors such as societal norms affect how individuals react to their own and others actions involving risk and uncertainty. This could be an important factor manipulating creative output. For instance, in some cultures, questioning authority is often suppressed and all forms of risk taking (calculated or otherwise) are discouraged when compared to the others. Future studies could investigate the extent to which cultural differences affect both risk taking and creativity.

## Conclusion

Our study demonstrates that individuals who possess a creative personality and mind-set are more likely to take risks exclusively in the social domain. These results thus highlight the importance of the role social risk taking attitudes play in creativity. The current research also emphasizes the need to investigate risk taking in a domain specific context. In our understanding, this is the first study to show that not only is creativity linked to risk taking, but also that this relationship is highly domain specific.

## Authors Contributions

All authors contributed to the design of the study, analysis or interpretation of the data. VT wrote the manuscript, YH, SH, MR, and SD provided critical inputs. All authors gave the final approval to the current version of the manuscript.

## Conflict of Interest Statement

The authors declare that the research was conducted in the absence of any commercial or financial relationships that could be construed as a potential conflict of interest. The reviewer NC and the handling Editor declared their shared affiliation, and the handling Editor states that the process nevertheless met the standards of a fair and objective review.

## Footnotes

1 www.surveymonkey.com
